# Guidelines for Guidelines: Are They Up to the Task? A Comparative Assessment of Clinical Practice Guideline Development Handbooks

**DOI:** 10.1371/journal.pone.0049864

**Published:** 2012-11-26

**Authors:** Shabnam Ansari, Arash Rashidian

**Affiliations:** 1 Students’ Scientific Research Center, School of Medicine, Tehran University of Medical Sciences, Tehran, Iran; 2 Department of Health Management and Economics, School of Public Health, Tehran University of Medical Sciences, Tehran, Iran; 3 Deputy Directors for Research, Knowledge Utilization Research Center, Tehran University of Medical Sciences, Tehran, Iran; Old Dominion University, United States of America

## Abstract

**Objectives:**

We conducted a comparative review of clinical practice guideline development handbooks. We aimed to identify the main guideline development tasks, assign weights to the importance of each task using expert opinions and identify the handbooks that provided a comprehensive coverage of the tasks.

**Methods:**

We systematically searched and included handbooks published (in English language) by national, international or professional bodies responsible for evidenced-based guideline development. We reviewed the handbooks to identify the main guideline development tasks and scored each handbook for each task from 0 (the handbook did not mention the task) to 2 (the task suitably addressed and explained), and calculated a weighted score for each handbook. The tasks included in over 75% of the handbooks were considered as ‘necessary’ tasks.

**Result:**

Nineteen guideline development handbooks and twenty seven main tasks were identified. The guideline handbooks’ weighted scores ranged from 100 to 220. Four handbooks scored over 80% of the maximum possible score, developed by the National Institute for Health and Clinical Excellence, Swiss Centre for International Health, Scottish Intercollegiate Guidelines Network and World Health Organization. Necessary tasks were: selecting the guideline topic, determining the guideline scope, identifying relevant existing guidelines, involving the consumers, forming guideline development group,, developing clinical questions, systematic search for evidence, selecting relevant evidence, appraising identifies research evidence, making group decision, grading available evidence, creating recommendations, final stakeholder consultation, guideline implementation strategies, updating recommendations and correcting potential errors.

**Discussion:**

Adequate details for evidence based development of guidelines were still lacking from many handbooks. The tasks relevant to ethical issues and piloting were missing in most handbooks. The findings help decision makers in identifying the necessary tasks for guideline development, provide an updated comparative list of guideline development handbooks, and provide a checklist to assess the comprehensiveness of guideline development processes.

## Introduction

The Institute of Medicine, in 1990, defined the clinical practice guidelines as ‘systematically developed statements to assist practitioner and patient decisions about appropriate health care for specific clinical circumstances [1]. Most recently it revised its definition to reflect the importance developing guidelines that are ‘informed by a systematic review of evidence and an assessment of the benefits and harms of alternative care options’ [2]. Guidelines are sought for improving the quality of care provided to the patients, reducing variability and containing the health care costs [3], [4]. Achieving those goals is difficult, and despite the development of clinical guidelines in many countries, solving the problems of cost and quality as well as variation in care remains a challenge to health systems.

Several countries have adopted the pro-active policies of guidelines development at the national level. These national programs are developed in response to the perceived (and observed) uncertainties in the quality of the published guidelines [5], [6], [7]. Developing valid clinical guidelines involves following a multi-stage program including several tasks, and each stage and task may be influenced by different biases [2], [8], [9]. National programs are also sought with the expectation that it will be easier and more fruitful to implement national guidelines than local or society developed clinical guidelines. Especially as the abundance of the guidelines developed by pharmaceutical companies, medical societies, local health authorities and interested groups of clinicians and academics has made it difficult for the practitioners to select and follow credible guidelines that are relevant to their practices [10].

The first national programs of guideline development started in the USA in the 1980s and in several other high income countries in the 1990s [11]. Most of the guideline development programs are established in high-income countries, where there are more (human and financial) resources available to the health systems. The progress in developing such national programs in low and middle income countries is still lagging behind. The WHO and other international organizations have also developed ad-hoc guidelines, as well as systematic guideline development programs [12].

The methodologies followed in guideline development programs varies. Burgers et al. (2003) conducted a survey on 18 clinical guideline programs produced in the USA, Canada, Australia and 9 European countries. They observed that the more recent programs were benefitting from the methodology created by the older ones. They also recommended that the programs should put further emphasis on the dissemination and implementation of the guidelines [13]. Van der Wees et al. compared six guideline programs against the Appraisal of Guidelines for Research and Evaluation (AGREE) criteria to update the Dutch program for guidelines in physical therapy [11], [14]. Similarly, Turner et al. assessed a limited number of six guideline development handbooks against the AGREE criteria for guideline development [15]. Turner et al. covered handbooks developed by the Council of Europe, the National Health and Medical Research Council of Australia, the National Institute for Health and Clinical Excellence in the UK, the New Zealand Guidelines Group, the Scottish Intercollegiate Guideline Network, and the World Health Organization [15].

As part of a wider initiative to design a program for the development of clinical guidelines at a national level in Iran we assessed the methods and approaches adopted by established guideline development programs around the world [16]. This study in a way is an update and expansion of the Turner et al. study. Two reasons convinced us to conduct the study. First, they had focused on a limited number of ‘tasks’ and not all the relevant tasks were included. Also only few handbooks were assessed in their study. Second, their study might not account for the recent advancements in guideline development techniques. In this study we both expanded the number of programs covered and the methods used for the assessment. Our aims were to identify the main guideline development tasks, assign weights to the importance of each task using expert opinions, and identify the handbooks that provided a comprehensive coverage of the tasks.

## Methods

### Selecting Guideline Development Handbooks

We systematically searched Pubmed and TRIP databases using general sensitive terms representing ‘clinical guideline development handbooks’. We also searched the Google using similar terms, aiming for the most popular handbooks (i.e. appearing on the first ten pages of Google search outputs) and literature published in English language and available on the net. We also contacted the experts and searched the reference lists of the identified literature and handbooks.

We included documents (‘handbooks’) produced by national or international organizations responsible for clinical guideline development and also professional and academic bodies working on guideline development. These handbooks may have been produced for the purpose of developing clinical guidelines in general, or targeting guidelines for specific clinical conditions. We only included those handbooks that were focused on the development of evidence-based guidelines.

### Identifying the Main Tasks for Guideline Development

Initially, we had planned to extract the main tasks of guideline development from NICE handbook [17], and then compare other handbooks to assess whether those guideline development tasks were covered. However, an initial screen of a few handbooks demonstrated that NICE handbook did not specify all the tasks of evidence-based guideline development (e.g. adapting existing guidelines was not included).

To determine other tasks not specified in NICE handbook, we first screened fifteen identified guideline development handbooks and designed an inclusive list of the tasks. The list was discussed and revised in a few meetings to ensure consensus was achieved. Other relevant ‘tasks’ were added as a result of elaborating on the task list (e.g. ethical issues). Finally, the tasks were re-ordered to ensure a logical flow existed, and in cases, re-phrased to reduce potential misinterpretations.

### Weighing the Importance of Guideline Development Tasks using Expert Opinions

We prepared a web-based questionnaire (using Google Document), and invited nineteen experts in the field (external to the research team) from seven different countries to respond to the questionnaire. We asked the participants to weigh each guideline development task on a 6-point scale from 0 (not important) to 5 (very high importance). We then used the median of the scores given to each task as the task ‘weight score’.

### Scoring Guideline Development Handbooks

Then two authors independently reviewed each handbook and compared its elements against the task list. Each handbook was scored for each task from 0 to 2 based on the following criteria:

- 2, if the handbooks addressed the task and provided enough information to suggest the task was given serious attention
- 1, if the handbook briefly (or just) mentioned the task
- 0, if the handbook did not appropriately mention the task

All disagreements about the scores were resolved through discussion between the authors. We considered, a priori, any task that was at least mentioned (i.e. scoring 1 or 2) in 75% of the handbooks was a ‘necessary’ task for evidence-based guideline development. We also considered the tasks as ‘relevant’ if they were mentioned in a minimum of three handbooks.

Then the scores given to each task for each guideline was multiplied with the task weight scores, and summed up to calculate the guideline’s total score. For each guideline we also calculated the percentage of the maximum score that was achieved by the guideline.

## Results

In total twenty seven main guideline development ‘tasks’ were noted and considered in this review (Table 1). Nineteen experts were approached using a web-based questionnaire to ‘weight’ the tasks, and twelve (63%) completed the questionnaire. They weighted each task using a 1 to 5 scoring system. The median of the scores given to the tasks ranged from 3 to 5. ‘Conducting economic evaluation’ scored the lowest weight score of 3. One the third of the tasks scored the highest score of 5 (Table 1).

**Table 1:** Guideline development tasks, their weight scores, and their coverage in the guideline development handbooks.

Task	Definition	Weight score	No. handbooks mentioning the task (%)
*Selecting the guideline topic*	The process and criteria for selecting and prioritizing topics	4.5	19 (100%)
*Determining the guideline scope*	A framework that describes the epidemiology of the disease or condition and theaspects of care and the settings is covered by the guideline	5	17 (89.4%)
*Preparing the work plan*	Specifying the guideline development project plan including timelinesand project costs	4	12 (63.1%)
*Identifying relevant existing guidelines*	An objective search of important and relevant databases and search enginesfor existing guidelines	4	15 (78.9%)
*Appraising relevant existing guidelines*	Objective appraisal of existing guidelines e.g. byusing AGREE	4	13 (68.4%)
*Adapting existing guidelines*	Describing guideline adaption methods	4	14 (73.6%)
*Involving consumers*	Contribution of the target population (patients, public, etc.) inrelevant tasks	4	17 (89.4%)
*Forming guideline development group*	Describing the composition of guideline development group, includingall relevant stakeholders	5	19 (100%)
*Managing conflict of interests*	Declaration of guideline development group memberscompeting interests	4.5	14 (73.6%)
*Running guideline development group*	Describing how to run a GDG (meetings, agenda items, chairing,responsibilities and roles)	5	14 (73.6%)
*Developing clinical questions*	Developing clinical question according to an objective approach,e.g. PICO framework	5	17 (89.4%)
*Systematic search for evidence*	Systematic searches of important bibliographicdatabases using sensitive key words	5	15 (78.9%)
*Selecting relevant evidence*	Inclusion and exclusion criteria for selecting theevidence	4.5	17 (89.4%)
*Appraising identified research evidence*	Appraising identified evidences using objective instruments(for example CASP tools)	5	18 (94.7%)
*Evidence synthesis and analysis*	Describing synthesis approaches of primary studies,including meta-analysis etc.	5	13 (68.4%)
*Conducting economic evaluation*	Describing the process of identifying, selecting andsynthesizing economic evaluation data	3	14 (73.6%)
*Making group decisions*	Using clear and objective consensus developmenttechniques (e.g. voting, Delphi,…)	4	17 (89.4%)
*Grading available evidence*	Appraising and summarizing the quality and strengthof recommendations	4	18 (94.7%)
*Considering ethical issues*	Discussing the approaches used for consideringethical issues in the guideline development process	4	7 (36.8%)
*Creating recommendations*	Interpreting the evidence to make recommendationsand the wording and format of recommendations	5	19 (100%)
*Final stakeholder consultation*	Final consultation with stakeholders beforepublishing the guideline	4	18 (94.7%)
*Publishing formats*	Describing different publication formats (fullguideline, quick reference guides, information for patient, wed-based publication, …)	4	14 (73.6%)
*Guideline implementation strategies*	Describing how the recommendations can be put intoPractice	4.5	19 (100%)
*Piloting*	Describing a process of pre-testing a guideline in thefield before its final release	4	9 (47.3%)
*Assessment the potential impacts of guideline implementation*	The cost and resource implications of implementingthe guideline in practice	4	13 (68.4%)
*Developing clinical audit and* *evaluation criteria*	Describing monitoring and auditing criteria andindicators to assess guideline implementation	4	14 (73.6%)
*Updating recommendations and correcting potential errors*	Describes the process, timeline, frequency andcriteria for updating recommendations or correcting errors	5	18 (94.7%)

We asked the experts about any other tasks that should have been included in the list. Four experts offered additional suggestions. Some of the suggestions had been adequately addressed in the task list (Table 2).

**Table 2:** Potential additional tasks for guideline development.

Experts’ suggestions: 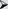 “Making recommendations sufficiently specific that they are useable in practice.” 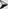 “For involving consumers, I think it may be better to say ‘involving stakeholders’, as consumers are one of a range of stakeholder groups; (2) Would add ‘identifying and appraising evidence on the feasibility and acceptability of the interventions’; (3) Would add ‘using a systematic and transparent approach to move from evidence to recommendations’; (4) Would add ‘appropriate organization and facilitation of the guideline panel meeting/s’. 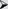 “I think you need a question about how far do you look for evidence - for examples is it important to look for all levels of evidence? Or within limited resources is it acceptable to limit the search to certain levels of evidence.” 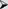 “Writing the guideline in different formats.” Additional tasks noted in the handbooks: 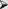 Requisition and use of local data in guideline development (in NZGG handbook [26]) 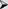 Using qualitative evidence as source of evidence for generating recommendations (in NZGG handbook [26]) 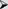 Considering equity issues in guideline development (in ACHR handbook [45])

We identified nineteen guideline development handbooks published in English language (Table 3). Twelve handbooks were developed by national guideline development programs or national associations. Others had international or regional mandates. We also identified four other potentially relevant handbooks that we did not include in the study for different reasons. Two handbooks almost totally tallied other handbooks already included in the review [18], [19] and two others provided too little info to make a meaningful assessment of the handbooks [20], [21].

**Table 3:** General description of the guideline development handbooks.

Handbook	Publication year	country of origin	General audience or targeting specific diseases	Website
National Institute for Health and Clinical Excellence (NICE)	2009	UK	General	www.nice.org.uk
Swiss Centre for International Health (SCIH)	2011	Swiss	General	http://www.swisstph.ch/
Scottish Intercollegiate Guidelines Network (SIGN)	2008	Scotland	General	www.sign.ac.uk
World Health Organization (WHO)	2012	International	General	www.who.int
Canadian Medical Association (CMA)	2007	Canada	General	www.accesscopyright.ca
New Zealand Guidelines Group (NZGG)	2001	New Zealand	General	www.nzgg.org.nz
National Health and Medical Research Council (NHMRC)	1998	Australia	General	www.health.gov.au/nhmrc
American Society of Clinical Oncology (ASCO)	2011	USA	Specific	www.asco.org
The Chartered Society of Physiotherapy (CSP)	2006	UK	Specific	www.csp.org.uk
International Diabetes Federation (IDF)	2003	International	Specific	www.idf.org
Advisory Committee on Health Research (ACHR)	2006	International	General	www.who.int/rpc/advisory_committee
World Stroke Organization (WSO)	2009	International	Specific	www.world-stroke.org
Cancer Care Ontario (CCO)	2011	Canada	Specific	www.cancercare.on.ca
Council of Europe (CE)	2001	International	General	www.social.coe.int
U.S. Preventive Services Task Force (UPSTF)	2008	USA	General	www.preventiveservices.ahrq.gov
Australian Health Policy Institute (AHPI)	2008	Australia	Specific	healthpolicystudies.org.au/
Regional Centre for Quality of Health Care (RCQHC)	2003	Regional	Specific	www.RCQHC.org
Royal College of Psychiatrists (RCP)	1994	UK	Specific	www.rcpsych.ac.uk
World Confederation for Physical Therapy (WCPT)	2006	International	Specific	www.wcpt.org

The handbooks were published within the period from 1994 to 2012. The guideline development handbooks’ raw scores ranged from 22 to 50 (out of a maximum of 54) and the weighted scores ranged from 100 to 220 (out of a maximum of 236). Four handbooks achieved over 80% of the maximum possible score NICE [17], SCIH [22], SIGN [23], WHO [24]. These were followed by the handbooks developed by CMA [25], NZGG [26], NHMRC [27], ASCO [28], CSP [29] and IDF [30], in order of their weighted scores (Table 4). Weighing the scores resulted in some changes in the ranking order of the handbooks, but the order of the handbooks ranked 1 to 4 remained unchanged.

**Table 4:** Guideline development handbooks raw and weighted scores.

Handbook	Raw score	Weighted score	Percentage of maximumweighted score
**National Institute for Health and Clinical Excellence (NICE) ** [**17**]	50	220	93.2
**Swiss Centre for International Health (SCIH) ** [**22**]	47	208	88.1
**Scottish Intercollegiate Guidelines Network (SIGN) ** [**23**]	45	199	84.3
**World Health Organization (WHO) ** [**24**]	44	195	82.6
**Canadian Medical Association (CMA) ** [**25**]	42	188	79.6
**New Zealand Guidelines Group (NZGG) ** [**26**]	42	187	79.2
**National Health and Medical Research Council (NHMRC) ** [**27**]	42	182.5	77.3
**American Society of Clinical Oncology (ASCO) ** [**28**]	41	182.5	77.3
**The Chartered Society of Physiotherapy (CSP) ** [**29**]	39	175.5	74.3
**International Diabetes Federation (IDF) ** [**30**]	40	173	73.3
**Advisory Committee on Health Research (ACHR) ** [**32**]	38	167	70.7
**World Stroke Organization (WSO) ** [**40**]	36	160.5	68
**Cancer Care Ontario (CCO) ** [**41**]	35	157.5	66.7
**Council of Europe (CE) ** [**31**]	36	154	65.2
**U.S. Preventive Services Task Force (UPSTF) ** [**42**]	33	149.5	63.3
**Australian Health Policy Institute (AHPI) ** [**43**]	28	128	54.2
**Regional Centre for Quality of Health Care (RCQHC) ** [**35**]	29	124.5	52.7
**Royal College of Psychiatrists (RCP) ** [**44**]	28	123.5	52.3
**World Confederation for Physical Therapy (WCPT) ** [**33**]	22	100.5	42.5

All the tasks were mentioned in at least three handbooks (Table 1). We defined the tasks mentioned by 75% of the handbooks, as ‘necessary’ tasks. Based on this definition, ‘selecting the guideline topic’, ‘determining the guideline scope’, ‘identifying relevant existing guidelines’, ‘involving the consumers’, ‘forming a guideline development group’, ‘developing clinical questions’, ‘systematic search for evidence’, ‘selecting relevant evidence from the searches’, ‘appraising identified research evidence’, ‘making group decision and reaching consensus’, ‘grading available evidence’, ‘creating recommendations’, ‘final stakeholder consultation’, ‘guidelines implementation strategies’, ‘updating recommendations and correcting potential errors’ are the necessary tasks for guideline development. We used the handbooks to produce brief narrative description of all the tasks, and provided a suggested reading list to note the handbooks that had provided practical and detailed explanations for certain tasks (Table 5). Also three further tasks were identified, each noted in one handbook only (Table 2).

**Table 5:** Suggested readings for detailed explanation of selected guideline development tasks.*

Task	Handbook
Selecting the guideline topic	SIGN, NZGG, CSP, ACHR, UPSTF
Determining the guideline scope	WHO
Adapting relevant existing guidelines	ACHR
Involving consumers (patients, …)	SIGN, NZGG
Forming guideline development group	WHO
Managing conflict of interests	WHO, ACHR
Running guideline development group	SIGN
Developing clinical questions	NZGG, UPSTF
Systematic search for evidence	NZGG
Selecting relevant evidence from the search results	UPSTF
Appraising identified research evidence	WHO, NZGG, UPSTF
Evidence synthesis and analysis	ASCO
Consider ethical issues	ACHR
Creating recommendation	SIGN, NZGG, ASCO, UPSTF
Guideline implementation strategies	SIGN, NHMRC, ASCO
Piloting the developed guideline	RCQHC

* The handbooks developed by NICE and SCIH provide a generally comprehensive guidance for guideline development. In this table we only note additional suggestions for further reading.

### Guideline Development Tasks

*Selecting the guideline topic* was addressed in all of the handbooks. For example the CE handbook stated: “prioritization of guideline topics may be based on the epidemiology of health problems, health inequalities, variations in the provision and quality of care, emergence of new technologies, or other factors that create a need for high quality, updated information” [31]. Previous versions of the NICE handbook did not mention this task, because selecting the topic was out of NICE’s mandate and the “topics were selected by the Department of Health” [17]. In its latest version, it now covers ‘selecting the topic’ as a guideline development task.

Seventeen of the nineteen handbooks mentioned *determining the guideline scope*, and thirteen handbooks provided enough information for a clinical guideline developer to understand how to complete that task. The NICE handbook asserted that “the scope provides a framework within which to conduct the development work. Its content briefly describes the background epidemiology relevant to the disease or condition and defines the aspects of care that the guideline will cover in terms of: population to be included or excluded, healthcare setting, interventions and treatments to be included and excluded.” [17].

Eight handbooks provided clear guidance on the way in which the *work plan* should be prepared. Four handbooks just mentioned the task; the rest did not address it. “A key step in the conduct of ASCO guidelines is completion of the clinical practice guideline development protocol worksheet. The worksheet specifies the purpose of the guideline, the target patient population and clinical outcomes of interest, key features of the systematic literature review, and a proposed timeline for guideline completion.” [28].

*Identifying relevant existing guidelines* was mentioned in fifteen handbooks, *appraising relevant existing guidelines* in thirteen handbooks, and *adapting relevant existing guidelines* in fourteen handbooks. ACHR states “in addition to supporting appropriate adaptation of its own guidelines, WHO should consider adapting guidelines developed by other organizations, given the potential value of WHO endorsement and savings, if high quality guidelines already exist … given WHO’s mandate; limited resources that are available to develop high quality guidelines that are informed by the best available evidence, particularly in low and middle income countries; and the potential to reduce unnecessary duplication, WHO should continue to develop international guidelines. However, these guidelines will often require adaptation and tailoring to local contexts:” [32].

All except two handbooks addressed *involving consumers* in some way, and ten handbooks provided clear guidance. To clarify the objectives and benefits of involving consumers, NZGG handbook stated: “the following objectives are needed to achieve the guiding principles: partnership and collaboration, democratic participation, equity and fairness, accountability, acceptability, to ensure the rights of consumers are upheld, to ensure consumer input is valued” [26].

*Forming a guideline development group* was mentioned in all of the handbooks and most had clear guidance, but they varied in their proposed methods for selecting the members, size of the group etc. As SCIH handbook focused on the development of health system guidance, it recommended the following composition for the guidance development groups: “expert(s) in health systems, expert(s) in the topic of the guidance, expert(s) in research synthesis and in knowledge translation, representatives of stakeholders, representative of potential users” [22].

Fourteen handbooks addressed managing *conflict of interests* and all fourteen handbooks explained it clearly except one. For example: “all experts participating in WHO meetings must declare any interest relevant to the meeting before their participation. In the case of guideline development this means that all members of the guideline development group and the expert review panel, as well as any other experts or advisers invited to guideline development meetings, should fill in a declaration of interests form.” [24].

*Running guideline development group* is mentioned by fourteen handbooks and twelve handbooks offered clear guidance. The NICE handbook stated: “running the guideline development group is the responsibility of the national collaborating center, in consultation with the Chair. Core responsibilities for all meetings include: setting meeting dates, planning agenda items, sending out papers, keeping records of all meetings and ensuring that all GDG members have a copy of the current guidelines manual” [17].

Five handbooks just mentioned *developing questions* and twelve handbooks described it in detail. NICE, SCIH, SIGN and WHO provided clear guidance on how to format questions in a patient, intervention, comparison and outcome (known as ‘PICO’) framework.

Fifteen handbooks addressed *systematic search for evidence* to gather the required information. Thirteen of them clearly mentioned developing search strategies based on questions, sources, and filters, and using stakeholder’s recommendations, although they varied in the provided detail.

*Selecting relevant evidence from the search results* was addressed by seventeen handbooks and ten handbooks explained it in detail. NICE handbook suggested: “first, the titles of the retrieved citations should be scanned and those that fall outside the topic of the guideline should be excluded. Next, the remaining abstracts should be scrutinized against the inclusion criteria agreed by the GDG” [17].

*Appraising identified research evidence* was addressed by eighteen handbooks and only one handbook did not even mention it. NICE and SIGN recommend the checklists developed originally by the Method for Evaluating Research and Guideline Evidence group in Australia and modified by SIGN. NZGG and IDF recommended using the GATE Notes (Generic Appraisal Tool for Epidemiology) developed by the Effective Practice Institute, University of Auckland to appraise the evidence. Word Confederation for Physical Therapy handbook suggested: “the Cochrane Collaboration produces robust systematic reviews and meta-analyses that can save a lot of work in assessing the literature” [33].

Thirteen handbooks mentioned *evidence synthesis and analysis,* and eight handbooks had clear guidance. ASCO handbook stated: “in putting together a practice guideline for a specific intervention or set of interventions, an ASCO expert panel may be faced with either interpreting existing meta-analyses or initiating a meta-analysis of its own. It is important to emphasize that not all systematic reviews will justify or require a formal meta-analysis. … The decision of whether or not to combine different studies in a meta-analysis is initially a clinical one, not a statistical one. The panel should determine, before deciding on the statistical approach, whether or not it makes sense to include within the same analysis patients who are different … or treatments that are different” [28].

Fourteen of nineteen handbooks mentioned *conducting economic evaluation* in guideline development, but only seven handbooks provided clear guidance on how to conduct it. According to NICE handbook: “economic evaluation will usually be conducted in the form of a cost-effectiveness analysis, with the health effects being measured using an appropriate non-monetary outcome indicator. In circumstances for which cost-effectiveness analysis is not appropriate, other validated methods may be used” [17]. The NZGG advised for developing a balance sheet to cover the costs and benefits of the recommendations: “a balance sheet is a formal itemization of the major costs and health benefits of a healthcare. The balance sheet could be considered as a first step in a full economic evaluation” [26].

*Making group decisions and reaching consensus* was mentioned by seventeen handbooks and thirteen handbooks provided clear explanations about it. The NICE handbook described the use of focus groups and formal consensus methods (i.e. the Delphi technique, the nominal-group technique, the consensus-development conference) for making group decisions [17].

Eighteen handbooks addressed *grading available evidence* and thirteen described it clearly. SCIH handbook stated: “grading recommendations reflects ‘the extent to which we can be confident that the desirable effects of an intervention outweigh the undesirable effects’. Strong recommendations imply that they can be adopted in most circumstances while weak recommendations will need additional considerations (e.g. decision aids)” [22].

Seven handbooks mentioned *considering ethical issues* in guideline development. Only two handbooks provided reasonable discussions of the issue, in additional documents. The NICE handbook provided guidance on ethical issue in documents besides the guidelines manual [34]. The Council of Europe stated: “guidelines are produced and used in the complex environment of a health care system with its ethical, economic, legal and other aspects; these aspects need to be taken into consideration in each country” [31]. Royal College of Psychiatrists handbook encouraged that guidelines might be applied more effectively by considering ethical issues.

*Creating recommendation* was mentioned by all handbooks and sixteen handbooks offered well-defined guidance. SIGN used the concept of ‘considered judgment’ besides the evidence table for each key question: “under this heading, guideline development groups summaries their view of the total body of evidence covered by each evidence table” [23].

All handbooks addressed *final stakeholder consultation* on recommendation drafts except one handbook. Word Confederation for Physical Therapy handbook defined a validation phase, in which “the draft guidelines can be sent to potential users to test practicality and clarity, and how acceptable the recommendations are. Patients and stakeholders can also review the draft guideline. The comments should be used by the GDG to adjust the guideline” [33].

*Publishing formats* was mentioned in eighteen handbooks and fourteen explained this phase in detail. According to the NICE handbook: “four separate documents are published: the full guideline, the NICE guideline, a quick reference guide and ‘understanding NICE guidance’ (information for patients and careers)” [17]. Similarly every SIGN guideline was published as a quick reference guide, in electronic format, and as information for patient and career.

All of the handbooks tackled *guideline implementation strategies*. Thirteen handbooks provided relatively detailed strategies to implement guidelines. SCIH handbook defines: “guidance implementation includes all those activities or strategies that lead to guidance actually being used in real life situations (measured, for example, by endorsers adherence to guidance recommendations)” [22].

Nine handbooks mentioned *piloting*, but only RCQHC handbook provided explanation on how to conduct the pilot phase and offered practical options [35]. SIGN handbook summarized the mood of many guideline development programs by asserting that: “the AGREE instrument suggested that guidelines should be pilot-tested prior to publication. SIGN considers that the pilot-testing phase is more appropriately carried out at local level” [23].

Thirteen handbooks considered *assessment of the potential impacts of guideline implementation* in guideline development process. According to NHMRC “it is important that the impact of guidelines is assessed. These assessments require complex study designs and will need to be carried out by researchers in collaboration with clinicians, rather than by clinicians alone” [27]. It suggested two potential methods for evaluation of guideline impact.

*Developing clinical audit and evaluation criteria* was mentioned in fourteen handbooks and eleven provided clear guidance on it.

All the handbooks except one addressed *updating and correcting potential errors* after guideline publication. Most of them offered defined updating processes. The IDF handbook stated: “there needs to be a statement of intention as to updating the guideline. If there is an umbrella organization to provide continuity, then it may be possible to specify a date, and plans should be made accordingly. Within the guideline it may be helpful to caution over some recommendations if major new evidence is expected in the near future.” [30].

## Discussion

We identified twenty seven tasks that should be considered for guideline development. These twenty seven tasks include the major elements of an evidence-based development process for clinical guidelines and can be used as a checklist for comparison of guideline development handbooks. We also noted a wide range of differences among guideline development handbooks. While some handbooks covered and referred to the majority of the tasks, no handbook on its own covered all the tasks. Fifteen tasks were included in at least 75% of the handbooks and we identified them as the ‘necessary tasks’ that should be covered in all guideline development programs. Furthermore, there was considerable variation in quality and depth of attention devoted to these tasks depending on the handbooks. Closer links and joint works on guideline development handbooks (e.g. via using Guidelines International Network forums) can be beneficial.

Compared to previous reviews [11], [13], [15], our study covers a wider collection of handbooks and provides an update on the current state of guideline development handbooks. Our study also benefits from a more comprehensive coverage of guideline development ‘tasks’. We also used the views of experts from several countries to weigh the tasks. Although the weighting process did not substantially change the results of the study, it acted as an external validation process for the task list. We organized the tasks in a logical order. However, the tasks do not exactly follow a step by step process. Involving consumers, for example, may exist in all stages of the guideline development process, and not as a specific stage in the process.

Among the tasks, the least amount of attention was devoted to *considering ethical issues* and *piloting*. There may be different reasons for such omissions. The *piloting* task may have been left out due to the technical difficulties of conducting a pilot on a guideline before its release. Already, the development of a clinical guideline is a time-consuming and laborious endeavor that usually takes around a year [36]. A proper pilot of a guideline can easily double this period. Given that many handbooks recommend an update of a guideline every two to three years [17], it is easily understandable why a pilot may not be feasible. There are, on the other hand, indispensible benefits for testing a guideline in the field before its release, and it may promote the guideline’s implementation which is the ultimate goal of developing guidelines [35]. Further work is required for the development of rapid pilot or field test approaches, and for assessing the efficiency of systematic field testing of newly developed guidelines.

Incorporating ethical concerns into guidelines remains a challenge. Unlike piloting (and to some extent, use of economic evaluation), where feasibility and capacity issues are the main barriers to their inclusion in guideline development, methodological and conceptual limitations seems to be the main barriers to formal consideration of ethical concerns in guideline development [37].

A further task that requires attention is incorporation of economic evaluation in guidelines. Five handbooks did not mention it at all and a further eight handbooks just briefly tapped on the issue, and only seven handbooks gave economic evaluation a decent attention. Also the experts gave the lowest weight score of 3 out of 5 to this task, implying the limited applications of the task in many guideline development programs. Guideline development programs should enhance their capacity for use of economic evaluation evidence and modeling in developing guidelines’ recommendations. It should be noted that even the guideline development entities that formally cherish use of economic evaluation evidence may find it difficult honoring their intention and ending up in patchy use of such evidence [38].

In addition to the twenty seven tasks noted above, we faced three further tasks suggested and explained in two handbooks (Table 2). Such tasks (e.g. using local data, obtaining qualitative evidence, considering equity issues) should be considered further as guideline development methods progress in future.

Obviously, the handbooks were not developed independently of each other but the links were not clear. SIGN, CSP and IDF handbooks referred to NICE handbook. On the other hand NICE, NHMRC, NZGG and CE handbooks mentioned the SIGN handbook. Closer links and joint works on guideline development handbooks can be beneficial.

Our study has some limitations. We only included documents that were published in English. There are other national guideline development programs (especially in Europe, also in Asia and Latin America) that may have not published the handbooks in English language. We included handbooks produced by national or international organizations as well as professional bodies working on clinical guideline development. As such the handbooks had different audiences. On the other hand the wider inclusion criteria helped us to include more guideline development handbooks.

We aimed to develop a comprehensive list of the tasks. However, depending on how detailed a task list might be, the list might include more or less number of tasks. The study was focused on ‘what’ written in the handbooks, and not what happened in reality, which might be better or worse than the handbooks. Despite these limitations, we reviewed a larger selection of handbooks using a more comprehensive list of tasks than Turner et al. and Van der Wees et al (who reviewed six handbooks each) [11], [15].

Turner et al. (2008) described fourteen key elements and compared the guidelines by these key elements. They concluded “the key elements of an evidence-based guideline development process are addressed with strong concordance by existing guideline development handbooks” [15]. Our findings challenge that conclusion. Burgers et al. (2003) sent questionnaires to eighteen guideline development programs. They noted that more recent programs were benefitting from methodology created by older programs and recommended that further emphasis should be devoted to guideline dissemination and implementation. Our findings suggest that despite the improvements, further work is required to ensure guideline development processes are up to the task of developing evidence based clinical guidelines.

This study helps health policy maker to compare guideline development handbooks and choose more suitable ones to use. It complements previous publications that focused on developing standard approaches for reporting clinical practice guidelines [39]. Also the tasks list can be used as a checklist to assess process of guideline development in any country. Further research is required to determine that clinical practice guideline development organizations are committed to follow the guideline development handbooks.
